# Supplementary material for: MetaQTL: a package of new computational methods for the meta-analysis of QTL mapping experiments
Source: BMC Bioinformatics. 2007 Feb 8;8:49. doi: 10.1186/1471-2105-8-49 (PMC1808479; doi:10.1186/1471-2105-8-49)
Supplement: Additional File 7 — MetaQTL Package : jar file and tutorial. This Zip archive contains both the MetaQTL JAR file and the files of the tutorial. [file 1471-2105-8-49-S7.zip › org.inra.metaqtl/doc/org/thalia/bio/entity/package-tree.html]

org.thalia.bio.entity Class Hierarchy


|  |  |  |
| --- | --- | --- |
| **Overview** \| **Package** \| Class \| Use \| **Tree** \| **Deprecated** \| **Index** \| **Help** | |  |
| **PREV**   **NEXT** | **FRAMES**    **NO FRAMES**     **All Classes** |  |


---


## Hierarchy For Package org.thalia.bio.entity

**Package Hierarchies:**: All Packages

---

## Class Hierarchy

- java.lang.Object
  - java.util.AbstractCollection<E> (implements java.util.Collection<E>)
    - java.util.AbstractList<E> (implements java.util.List<E>)
      - java.util.ArrayList<E> (implements java.lang.Cloneable, java.util.List<E>, java.util.RandomAccess, java.io.Serializable)
        - org.thalia.bio.entity.**BioEntityCollection** (implements java.util.Collection<E>, org.thalia.bio.IBioEntity)
    - org.thalia.bio.entity.**BioEntity** (implements org.thalia.bio.IBioEntity)
      - org.thalia.bio.entity.**BioEntityContainer** (implements org.thalia.bio.IBioEntity)
        - org.thalia.bio.entity.**AlleleContainer** (implements org.thalia.bio.IBioLocus)
          - org.thalia.bio.entity.**Locus** (implements org.thalia.bio.IBioLocus)
            - org.thalia.bio.entity.**Marker** (implements org.thalia.bio.IBioLocus)- org.thalia.bio.entity.**QTL** (implements org.thalia.bio.IBioLocus)
          - org.thalia.bio.entity.**GroupContainer** (implements org.thalia.bio.IBioGenome)
            - org.thalia.bio.entity.**GeneticMap**
            - org.thalia.bio.entity.**Individual**- org.thalia.bio.entity.**IndividualContainer** (implements org.thalia.bio.IBioPopulation)
                - org.thalia.bio.entity.**Population** (implements org.thalia.bio.IBioPopulation)
                  - org.thalia.bio.entity.**CrossPopulation** (implements org.thalia.bio.IBioCross)
                - org.thalia.bio.entity.**LocusContainer** (implements org.thalia.bio.IBioLGroup)
                  - org.thalia.bio.entity.**LGroup** (implements org.thalia.bio.IBioLGroup)
        - org.thalia.bio.entity.**Ontology** (implements org.thalia.bio.IBioOntology)- org.thalia.bio.entity.**OntologyTerm** (implements org.thalia.bio.IBioOntologyTerm)

## Interface Hierarchy

- org.thalia.bio.entity.**IBioConstants**- org.thalia.bio.entity.**IGeneticMapProperties**

---


|  |  |  |
| --- | --- | --- |
| **Overview** \| **Package** \| Class \| Use \| **Tree** \| **Deprecated** \| **Index** \| **Help** | |  |
| **PREV**   **NEXT** | **FRAMES**    **NO FRAMES**     **All Classes** |  |


---
